# Detection of *Leishmania* sp. kDNA in questing *Ixodes ricinus* (Acari, Ixodidae) from the Emilia-Romagna Region in northeastern Italy

**DOI:** 10.1007/s00436-022-07655-9

**Published:** 2022-09-09

**Authors:** Alice Magri, Monica Caffara, Marialetizia Fioravanti, Roberta Galuppi

**Affiliations:** Department of Veterinary Medical Sciences – Alma Mater Studiorum, University of Bologna, Ozzano Emilia, Bologna, Italy

**Keywords:** Leishmaniasis, *Leishmania* sp., *Ixodes ricinus*, Ticks

## Abstract

To date, sand flies (Phlebotominae) are the only recognized biological vectors of *Leishmania infantum*, the causative agent of human visceral leishmaniasis, which is endemic in the Mediterranean basin and also widespread in Central and South America, the Middle East, and Central Asia. Dogs are the main domestic reservoir of zoonotic visceral leishmaniasis, and the role of secondary vectors such as ticks and fleas and particularly Rhipicephalus sanguineus (the brown dog tick) in transmitting *L. infantum* has been investigated. In the present paper, the presence of *Leishmania* DNA was investigated in questing *Ixodes ricinus* ticks collected from 4 rural areas included in three parks of the Emilia-Romagna Region (north-eastern Italy), where active foci of human visceral leishmaniasis have been identified. The analyses were performed on 236 DNA extracts from 7 females, 6 males, 72 nymph pools, and 151 larvae pools. Four samples (1.7%) (i.e., one larva pool, 2 nymph pools, and one adult male) tested positive for *Leishmania* kDNA. To the best of our knowledge, this is the first report of the presence of *Leishmania* kDNA in questing *I. ricinus* ticks collected from a rural environment. This finding in unfed larvae, nymphs, and adult male ticks supports the hypothesis that *L. infantum* can have both transstadial and transovarial passage in *I. ricinus* ticks. The potential role of *I. ricinus* ticks in the sylvatic cycle of leishmaniasis should be further investigated.

## Introduction

*Leishmania infantum* (Kinetoplastea, Trypanosomatida) is the causative agent of human visceral leishmaniasis, an important zoonosis endemic in the Mediterranean basin and also widespread in Central and South America, the Middle East, and Central Asia (Alvar et al. 2012). The parasite is naturally transmitted to humans by phlebotomine sand flies and, in the peridomestic cycle, the dog is traditionally recognized as a reservoir (Podaliri Vulpiani et al. 2011), for its high susceptibility to the infection and heavy skin parasitism (Dantas-Torres 2007).

Sand flies are the only recognized biological vectors for *L. infantum*, and their rapid geographical spread is followed by the spread of leishmaniasis into previously free areas (Dujardin et al. 2008), although secondary routes of transmission (i.e., transfusions, vertical in utero transmission, and venereal transmission) of little epidemiological relevance have been reported in dogs (de Freitas et al. 2006; Silva et al. 2009; Boggiatto et al. 2011). However, a possible role of secondary vectors such as ticks and fleas has been suggested (Coutinho et al. 2005; 2007). In particular, the brown dog tick *Rhipicephalus sanguineus* has received a lot of attention mainly due to its parasitic life cycle and its close relationship with dogs in both rural and urban areas, being highly adapted to live within human dwellings (Dantas-Torres 2010). Although a considerable amount of research has been carried out to investigate the presence of *L. infantum* in *R. sanguineus* collected from dogs (Colombo et al. 2010; Dantas-Torres et al. 2010a; Solano-Gallego et al. 2012; Campos and Costa 2014; Medeiros-Silva et al. 2015) and possible transmission routes (e.g., ticks bites, ingestion of infected ticks) (McKenzie 1984; Coutinho et al. 2005), their role in the transmission of *L. infantum* has been debated (Otranto and Dantas-Torres 2010) and is still questioned. *L. infantum* DNA was also detected in the castor bean tick *Ixodes ricinus*: particularly, Trotta et al. (2012) found it in ticks collected from dogs in Central Italy, and subsequently Salvatore et al. (2014) detected it in *I. ricinus* from both dogs and cats in Northern Italy, areas where human visceral leishmaniasis is endemic. To the best of our knowledge, no previous research has been performed to establish the presence of *Leishmania* spp*.* in questing ticks from rural environments. The present study is focused on the search of *Leishmania* spp. in *I. ricinus* questing ticks collected from three parks of Emilia-Romagna region (northeastern Italy), in hilly areas where human visceral leishmaniasis has long been described.

## Material and methods

### Sampling

**Fig. 1:**
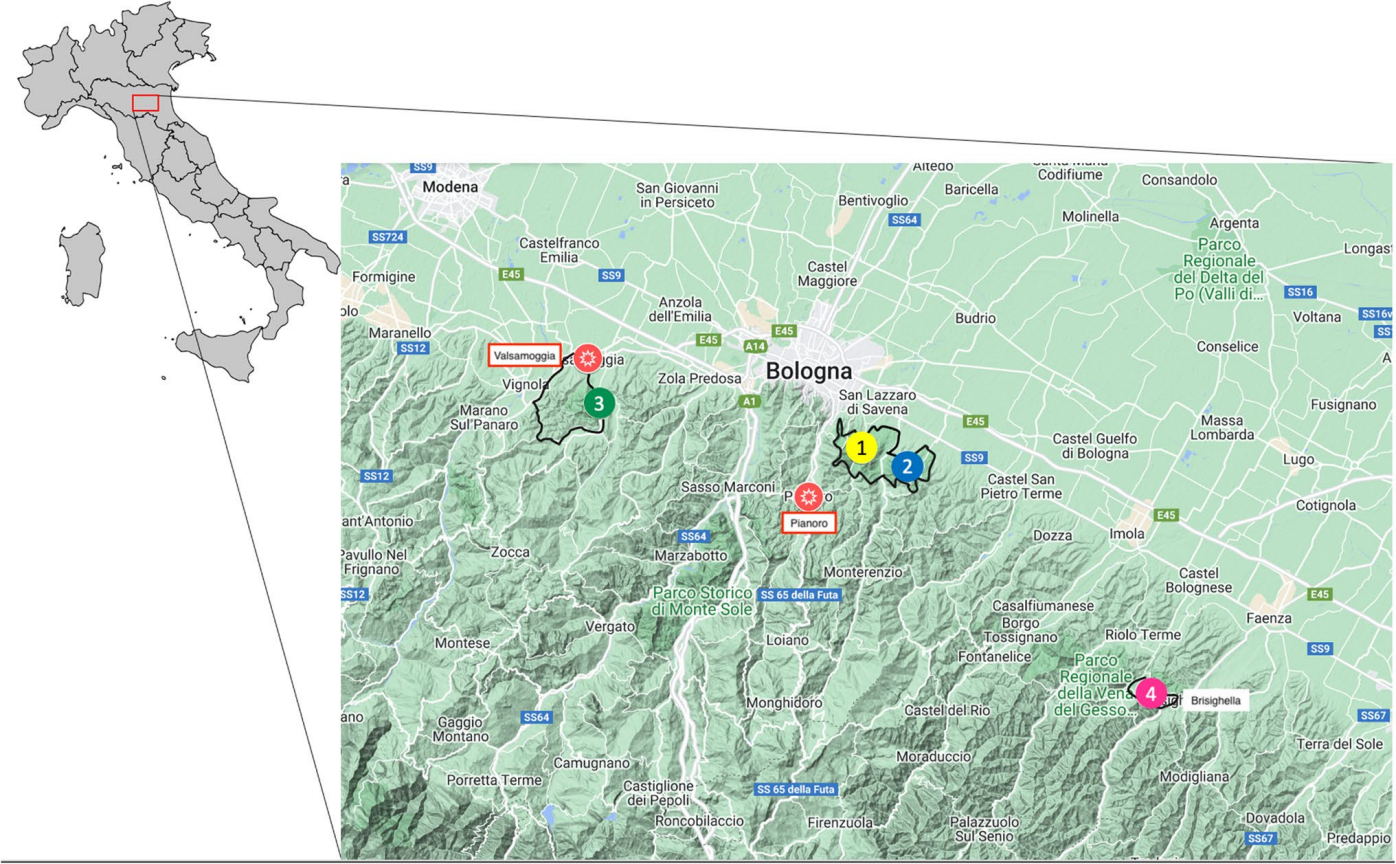
Map of the four sampling sites distributed in three parks of Emilia-Romagna region. Gessi Bolognesi and Calanchi dell’Abbadessa Park: “number 1 in yellow circle” Ca’ de Mandorli and “number 2 in blue circle” Ciagnano. Monteveglio Abbey, Park site: “number 3 in green circle”; Carnè Park site “number 4 in pink circle”. Park borders are marked with black lines. “Asterisk symbol in red circle”Active foci of human visceral leishmaniasis in Valsamoggia and Pianoro (VL single cases have been reported along the whole foothill side)

The analyses were performed on DNA extracts from questing *Ixodes ricinus* ticks collected from April to October 2010 in 4 sites within 3 parks of the Emilia-Romagna region (Fig. 1). The parks are located along the hilly area of the Apennines, where the presence of autochthonous cases of leishmaniasis has been described in both humans and dogs (Mollicone et al. 2003; Varani et al. 2013). The area is characterized by a series of gypsum outcrops, closed valleys, cliffs, forested mountains, and gray calanques alternated with farmland. The sampling sites are natural pathways and picnic areas with habitual human attendance. Questing ticks were collected every 15 days by continuously flagging with a 1 m^2^ white cotton cloth, from transects of 20 m along the uphill side of the pathways, usually reported as having higher tick density than the downhill side, while the picnic areas were flagged completely as described in detail by Aureli et al. (2015). Collected ticks were preserved in 70% ethanol at room temperature. Following morphological identification performed according to Manilla (1998) and Iori et al. (2005), ticks (individual adults, and pools consisting of either 5 nymphs or 10 larvae) were processed for DNA extraction as described by Aureli et al. (2015). Overall, 236 DNA extracts from 7 females, 6 males, 72 nymphs pools (i.e., 380 nymphs) and 151 larvae pools (i.e., 1510 larvae) were analyzed.

### DNA analysis

To detect the presence of *Leishmania* kDNA, a real-time PCR protocol was performed targeting a 71-bp region of the minicircle kinetoplast DNA using the primer pair Leish71Up (5′-CCAAACTTTTCTGGTCCTYCGGGTAG-3′) and Leish71Do (5′-TGAACGGGATTTCTGCACCCATTTTTC-3′) (Tsakmakidis et al. 2017) and following the conditions reported by Magri et al. (2022a, b).

## Results and discussion

Out of the 236 *I. ricinus* DNA extracts, 4 (1.7%) tested positive for *Leishmania* sp. in 2 of the four sites examined: 2 nymph pools (5.4%) and 1 adult male (33.3%) from Monteveglio Abbey Park and 1 larva pool (2.3%) from Carné Park (Table 1).

**Table 1:** Number of specimens examined according to sampling sites and developmental stages. T = total; P = positive

	Gessi Bolognesi and Calanchi dell’Abadessa Park									
	1 Cà de Mandorli		2 Ciagnano		3 Monteveglio Abbey Park		3 Carnè Park		Total	
	*T*	*P*	*T*	*P*	*T*	*P*	*T*	*P*	*T*	*P*
Adult females	3	0	1	0	3	0	0	-	7	0
Adult males	2	0	1	0	3	1 (33.3%) (95% *CI*: 0.0–53.3)	0	-	6	1 (16.6%) (95% *CI*: 0.0–46.37)
Nymphs (pools)	24	0	11	0	37	2 (5.4%) (95% *CI*: 0.0–12.7)	0	-	72	2 (2.8%) (95% *CI*: 0.0–6.61)
Larvae (pools)	13	0	74	0	21		43	1 (2.3%) (95% *CI*: 0.0–35.5)	151	1 (0.66%)
Total	42	0	87	0	64	3 (4.7%) (95% *CI*: 0.0–9.9)	43	1	236	4 (1.7%) (95% *CI*: 0.05–3.3)

*T* total; *P* positive

Previous research mainly investigated the brown dog tick as a possible vector of *L. infantum*, and several studies showed the presence of *L. infantum* DNA in *R. sanguineus* collected from dogs affected by canine leishmaniasis (Coutinho et al. 2005; Paz et al. 2010; Campos and Costa 2014; Medeiros-Silva et al. 2015; Viol et al 2016) and from seronegative dogs living in endemic areas (Solano-Gallego et al. 2012). Nevertheless, the finding of *Leishmania* DNA in *R. sanguineus* ticks could be expected, given their blood feeding habits. Further work speculated that brown ticks can transmit canine leishmaniasis: Dantas-Torres et al. (2010a) reported the presence of *L. infantum* kDNA in the salivary glands of *R. sanguineus* ticks, corroborating the hypothesis that ticks could inject *Leishmania* parasites while blood feeding. Colombo et al. (2010) found viable *Leishmania* by RNA analysis in ticks 7 to 10 days after their removal from the dogs, showing that the parasite could survive for a long period in ticks, even after ecdysis had occurred in laboratory conditions. Dantas-Torres et al (2010b) demonstrated the transovarial passage of *L. infantum* kDNA in artificially infected *R. sanguineus*, and a subsequent study (Dantas-Torres et al. 2011) reported the detection and quantification of *L. infantum* DNA in field-collected engorged females and in their eggs and larvae. The transstadial and transovarian transmission of *L. infantum* in *R. sanguineus* was further confirmed by Dabaghmanesh et al. (2016). Medeiros-Silva et al. (2015), isolated *Leishmania* spp. in cultures from salivary glands and intestines of ticks collected from dogs; interestingly, it was possible to culture the parasite also from pools of unfed male ticks suggesting that *Leishmania* could persist in the brown tick after blood digestion (Medeiros-Silva et al. 2015). *L. infantum* DNA was also reported from questing *Rhipicephalus* spp. from Israel (Mumcuoglu et al. 2022). Finally, further studies demonstrated the capability of *R. sanguineus* nourished on infected dogs to transmit the parasites to hamsters (Almeida et al. 2016). Based on these findings, although sand flies are the recognized vectors of *Leishmania,* a minor role of the dog brown tick could not be excluded.

Concerning tick species other than *R. sanguineus*, also *I. ricinus* collected from dogs in central Italy was found positive for *L. infantum* (Trotta et al. 2012). Moreover, Salvatore et al (2014) found *Leishmania* kDNA in *I. ricinus* ticks removed from 4 dogs and 1 cat living in areas of northeastern Italy where canine leishmaniasis is endemic, although no anamnestic data related to infection in these animals were reported.

In the Argentine Patagonia, Millan et al. (2016) observed the presence of *Leishmania* DNA in the gray fox *Pseudalopex griseus* and in pools of *Amblyomma tigrinum* ticks collected from both positive and negative foxes, in a remote non-endemic area of South America, where dogs are scarce and sand flies are not known to be present, supporting the hypothesis that *L. infantum* could maintain a sylvatic cycle in the studied area, not involving dogs or sand flies.

Interestingly, in the same areas where the present study was carried out, a high positivity rate for *L. infantum* (33.3%) was observed in the roe deer *Capreolus capreolus* (Magri et al. 2022a, b), and blood meal preference for this species was found in sand flies (Calzolari et al. 2022), suggesting the possible involvement of *C*. *capreolus* (frequently hosts of the adult stages of *I. ricinus*) in the epidemiology of leishmaniasis in the area under study.

## Conclusions

To the best of our knowledge, this is the first description of *Leishmania* DNA in questing *I. ricinus* ticks collected from a rural environment. This finding in unfed larvae, nymphs and males support the hypothesis that, even in this tick species, *L. infantum* could have both transstadial and transovarial transmission. The percentage (1.7%) of ticks positive to *Leishmania* DNA obtained in our study appears lower than the one reported in sand flies in other research (2.9–57.1%), stressing the fact that phlebotomine flies are the sole *Leishmania* efficient proven vector (Aransay et al. 2000; Gómez-Saladín et al. 2005; Ergunay et al. 2014; González et al. 2017; Latrofa et al. 2018). Nevertheless, a role of *I. ricinus* in a sylvatic cycle, albeit minor, could not be excluded in the endemic areas under study.

## Data Availability

Data supporting the conclusions of this article are included within the article and its supplementary tables.

## References

[CR1] Almeida VA, da Hora TN, Júnior NFL, Carvalho FS, da Silva AL, Wenceslau AA, Albuquerque GR, Silva FL (2016). Detection of *Leishmania infantum* DNA in hamsters infested with ticks collected from naturally infected dogs. Rev Bras Med Vet.

[CR2] Alvar J, Vélez ID, Bern C, Herrero M, Desjeux P, Cano J, Jannin J, den Boer M, WHO Leishmaniasis Control Team (2012). Leishmaniasis worldwide and global estimates of its incidence. PloS one.

[CR3] Aransay AM, Scoulica E, Tselentis Y (2000). Detection and identification of *Leishmania* DNA within naturally infected sand flies by seminested PCR on minicircle kinetoplastic DNA. Appl Environ Microbiol.

[CR4] Aureli S, Galuppi R, Ostanello F, Foley JE, Bonoli C, Rejmanek D, Rocchi G, Orlandi E, Tampieri MP (2015). Abundance of questing ticks and molecular evidence for pathogens in ticks in three parks of Emilia-Romagna region of Northern Italy. Ann Agric Environ Med.

[CR5] Boggiatto PM, Gibson-Corley KN, Metz K, Gallup JM, Hostetter JM, Mullin K, Petersen CA (2011). Transplacental transmission of Leishmania infantum as a means for continued disease incidence in North America. PLoS Negl Trop Dis.

[CR6] Calzolari M, Romeo G, Bergamini F, Dottori M, Rugna G, Carra E (2022). Host preference and *Leishmania infantum* natural infection of the sand fly *Phlebotomus perfiliewi* in northern Italy. Acta Trop.

[CR7] Campos JH, Costa FAL (2014). Participation of ticks in the infectious cycle of canine visceral Leishmaniasis in Teresina, Piauí, Brazil. Rev Inst Med Trop Sao Paulo.

[CR8] Colombo FA, Ororizzi RMFN, Laurenti MD, Galati EAB, Canavez F, Pereira-Chioccola VL (2010). Detection of *Leishmania (Leishmania) infantum* RNA in fleas and ticks collected from naturally infected dogs. Parasitol Res.

[CR9] Coutinho MT, Linardi PM (2007). Can fleas from dogs infected with canine visceral leishmaniasis transfer the infection to other mammals?. Vet Parasitol.

[CR10] Coutinho MTZ, Bueno LL, Sterzik A, Fujiwara RT, Botelho JR, De Maria M, Genaro O, Linardi PM (2005). Participation of *Rhipicephalus sanguineus* (Acari: Ixodidae) in the epidemiology of canine visceral leishmaniasis. Vet Parasitol.

[CR11] Dabaghmanesh T, Asgari Q, Moemenbellah-Fard MD, Soltani A, Azizi K (2016). Natural transovarial and transstadial transmission of *Leishmania infantum* by naïve *Rhipicephalus sanguineus* ticks blood feeding on an endemically infected dog in Shiraz, south of Iran. Tans R Soc Trop Med Hyg.

[CR12] Dantas-Torres F (2007). The role of dogs as reservoirs of *Leishmania* parasites, with emphasis on *Leishmania (Leishmania) infantum* and *Leishmania (Viannia) braziliensis*. Vet Parasitol.

[CR13] Dantas-Torres F (2010). Biology and ecology of the brown dog tick Rhipicephalus Sanguineus. Parasit Vectors.

[CR14] Dantas-Torres F, Lorusso V, Testini G, De Paiva-Cavalcanti M, Figueredo AL, Stanneck D, Mencke N, Brandão-Filho SP, Alved LC, Otranto D (2010). Detection of *Leishmania**infantum* in *Rhipicephalus** sanguineus* ticks from Brazil and Italy. Parasitol Res.

[CR15] Dantas-Torres F, Martins TF, de Paiva-Cavalcanti M, Figueredo LA, Lima BS, Brandão-Filho SP (2010). Transovarial passage of *Leishmania infantum* kDNA in artificially infected *Rhipicephalus sanguineus*. Exp Parasitol.

[CR16] Dantas-Torres F, Latrofa MS, Otranto D (2011). Quantification of *Leishmania infantum* DNA in females, eggs and larvae of *Rhipicephalus sanguineus*. Parasit Vectors.

[CR17] de Freitas E, Melo MN, da Costa-Val AP, Michalick MS (2006). Transmission of *Leishmania infantum* via blood transfusion in dogs: potential for infection and importance of clinical factors. Vet Parasitol.

[CR18] Dujardin JC, Campino L, Cañavate C, Dedet JP, Gradoni L, Soteriadou K, Mazeris A, Ozbel Y, Boelaert M (2008). Spread of vector-borne diseases and neglect of Leishmaniasis. Europe Emerg Infect Dis.

[CR19] Ergunay K, Kasap OE, Orsten S, Oter K, Gunay F, Yoldar AZ, Dincer E, Alten B, Ozkul A (2014). Phlebovirus and *Leishmania* detection in sandflies from eastern Thrace and northern Cyprus. Parasites Vectors.

[CR20] Gómez-Saladín E, Doud CW, Maroli M (2005). Short report: surveillance of *Leishmania* sp. among sand flies in Sicily (Italy) using a fluorogenic real-time polymerase chain reaction. Am J Trop Med Hyg.

[CR21] González E, Álvarez A, Ruiz S, Molina R, Jiménez M (2017). Detection of high *Leishmania infantum* loads in *Phlebotomus perniciosus* captured in the leishmaniasis focus of southwestern Madrid region (Spain) by real time PCR. Acta Trop.

[CR22] Iori A, Di Giulio A, De Felici S (2005) Zecche d’Italia. In: Cringoli G, Iori A, Rinaldi L, Veneziano V, Genchi C (eds.). Mappe parassitologiche: Zecche. Rolando Editore, Napoli 52–163

[CR23] Latrofa MS, Iatta R, Dantas-Torres F, Annoscia G, Gabrielli S, Pombi M, Gradoni L, Otranto D (2018). Detection of *Leishmania infantum* DNA in phlebotomine sand flies from ad area where canine leishmaniosis is endemic in southern Italy. Vet Parasitol.

[CR24] Magri A, Galuppi R, Fioravanti M, Caffara M (2022a) Survey on the presence of *Leishmania* sp. in peridomestic rodents from the Emilia‑Romagna Region (North‑Eastern Italy) Vet Res Commun. 10.1007/s11259-022-09925-4. 10.1007/s11259-022-09925-4 35412180

[CR25] Magri A, Bianchi C, Kostygov A, Caffara M, Galuppi R, Fioravanti M, Jurčenko V (2022b) Survey on the presence of *Leishmania infantum* in wild animals from the province of Bologna (North-eastern Italy). XXXII Congresso SoIPa – Naples (Italy), 27–30 June 2022b: 306

[CR26] Manilla G (1998) Fauna d’Italia. Acari Ixodida. Edizioni Calderini, Bologna, Italy

[CR27] McKenzie KK (1984) A study of the transmission of canine leishmaniasis by the tick, *Rhipicephalus sanguineus*, and an ultrastructural comparison of the promastigote. PhD Dissertation, Oklahoma State University

[CR28] Medeiros-Silva V, Gurgel-Gonçalves R, Nitz N, D’Anduraim Morales LE, Cruz LM, Sobrai IG, Boité MC, Ferreira GEM, Cupolillo E, Romero GAS (2015). Successful isolation of *Leishmania infantum* from *Rhipicephalus sanguineus* sensu lato (Acari: Ixodidae) collected from naturally infected dogs. BMC Vet Res.

[CR29] Millán J, Travaini A, Zanet S, López-Bao JV, Trisciuoglio A, Ferroglio E, Rodríguez A (2016). Detection of *Leishmania* DNA in wild foxes and associated ticks in Patagonia, Argentina, 2000 km south of its known distribution area. Parasites Vectors.

[CR30] Mollicone E, Battelli G, Gramiccia M, Maroli M, Baldelli R (2003). A stable focus of canine leishmaniosis in the Bologna Province, Italy. Parassitologia.

[CR31] Mumcuoglu KY, Arslan-Akveran GA, Aydogdu S, Karasartova D, Kosar A, Savci U, Keskin A, Taylan-Ozkan,  (2022). Pathogens in ticks collected in Israel: II Bacteria and Protozoa Found *in Rhipicephalus Sanguineus* Sensu Lato and *Rhipicephalus Turanicus*. Ticks Tick Borne Dis.

[CR32] Otranto D, Dantas-Torres F (2010). Fleas and ticks as vectors of *Leishmania* spp. to dogs: caution is needed. Vet Parasitol.

[CR33] Paz GF, Ribeiro MFB, de Magalhães DF, Sathler KPB, Morais MH, Fiúza VOP, Brandão ST, Werneck GL, Fortes-Dias CL, Dias ES (2010). Association between the prevalence of infestation by *Rhipicephalus sanguineus* and *Ctenocephalides felis felis* and the presence of anti-*Leishmania* antibodies: a case–control study in dogs from a Brazilian endemic area. Prev Vet Med.

[CR34] Podaliri Vulpiani M, Iannetti L, Paganico D, Iannino F, Ferri N (2011). Methods of control of the *Leishmania infantum* dog reservoir: state of the Art. Vet Med Int.

[CR35] Salvatore D, Aureli S, Baldelli R, di Francesco A, Tampieri MP, Galuppi R (2014) Molecular evidence of *Leishmania infantum* in *Ixodes ricinus* ticks from dogs and cats, in Italy. Vet Ital 50(4):307–312. 10.12834/VetIt.83.1222.2. 10.12834/VetIt.83.1222.2 25546069

[CR36] Silva FL, Oliveira RG, Silva TM, Xavier MN, Nascimento EF, Santos RL (2009). Venereal transmission of canine visceral leishmaniasis. Vet Parasitol.

[CR37] Solano-Gallego L, Rossi L, Scroccaro AM, Montarsi F, Caldin M, Furlanello T, Trotta M (2012). Detection of *Leishmania infantum* DNA mainly in *Rhipicephalus sanguineus* male ticks removed from dogs living in endemic areas of canine leishmaniosis. Parasites Vectors.

[CR38] Trotta M, Nicetto M, Fogliazza A, Montarsi F, Caldin M, Furlanello T, Solano-Galego L (2012). Detection of *Leishmania infantum*, *Babesia canis*, and rickettsiae in ticks removed from dogs living in Italy. Ticks Tick Borne Dis.

[CR39] Tsakmakidis I, Angelopoulou K, Dovas CI, Dokianakis E, Tamvakis A, Symeonidou I, Antoniou M, Diakou A (2017). *Leishmania* infection in rodents in Greece. Trop Med Int Health.

[CR40] Varani S, Cagarelli R, Melchionda F, Attard L, Salvadori C, Finarelli A, Gentilomi G, Tigani R, Rangoni R, Todeschini R, Scalone A, Di Muccio T, Gramiccia M, Gradoni L, Viale P, Landini M (2013). Ongoing outbreak of visceral leishmaniasis in Bologna Province, Italy, November 2012 to May 2013. Euro Surveill.

[CR41] Viol MA, Guerrero FD, de Oliveira BCM, de Aquino MCC, Loiola SH, de Melo GD, de Souza Gomes AH, Kanamura CT, Garcia MV, Andreotti R, de Lima VMF, Bresciani KDS (2016). Identification of *Leishmania* spp. promastigotes in the intestines, ovaries, and salivary glands of *Rhipicephalus sanguineus* actively infesting dogs. Parasitol Res.

